# Incidence and survival for oropharynx and non‐oropharynx head and neck cancers among veterans living with HIV

**DOI:** 10.1002/cam4.3539

**Published:** 2020-10-23

**Authors:** Angela L. Mazul, Christine Hartman, Jennifer Kramer, Donna L. White, Kathryn Royse, Suchismita Raychaudhury, Vlad Sandulache, Sarah T. Ahmed, Peter Richardson, Andrew G. Sikora, Elizabeth Chiao

**Affiliations:** ^1^ Department of Otolaryngology/Head and Neck Surgery Washington University School of Medicine Saint Louis MO USA; ^2^ Division of Public Health Science Department of Surgery Washington University School of Medicine Saint Louis MO USA; ^3^ VA Health Services Research Center of Innovations in Quality Effectiveness, and Safety (IQuESt Michael E. DeBakey VA Medical Center Houston TX USA; ^4^ Department of Medicine Baylor College of Medicine Houston TX USA; ^5^ Dan L Duncan Comprehensive Cancer Center Baylor College of Medicine Houston TX USA; ^6^ Bobby R. Alford Department of Otolaryngology‐Head and Neck Surgery Baylor College of Medicine Houston TX USA; ^7^ Otolaryngology Section Operative Care Line Michael E. DeBakey Veterans Affairs Medical Center Houston TX USA

**Keywords:** head and neck cancer, HIV, incidence, survival

## Abstract

**Background:**

People living with HIV/AIDS (PLWH) have an excess risk for head and neck squamous cell carcinoma (HNSCC) compared to the general U.S. population, but little is known about HIV‐specific risk factors associated with the incidence and outcomes HNSCC. We aim to identify clinical and HIV‐specific risk factors associated with oropharyngeal and non‐oropharyngeal HNSCC incidence and outcomes separately.

**Methods:**

We constructed a retrospective cohort study of 45,052 PLWH aged 18 or above from the national Veteran Affairs (VA) Corporate Data from 1999 to 2015. We extracted demographic data and risk factor information, including history of alcohol abuse, smoking, CD4 count (cells/μl), and percent of follow‐up time with undetectable HIV viral load as time‐updated variables. We calculated the age‐standardized incidence rates of oropharyngeal and non‐oropharyngeal HNSCC and estimated adjusted hazard ratios (HR). We also examined overall survival using Kaplan–Meier curves and adjusted HR.

**Results:**

The standardized incidence rate of oropharyngeal and non‐oropharyngeal HNSCC in this veteran cohort of PLWH is 23.0 (95% confidence intervals (CIs): 17.1‐28.9) and 55.4 (95% CI: 46.5‐64.3) per 100,000 person‐years, respectively. Nadir CD4 count ≤200 was associated with an increased risk of non‐oropharyngeal HNSCC (HR: 1.78; 95% CI: 1.31‐2.30 vs >200). Five‐year overall survival of OPSCC (37.0%) was significantly lower than non‐oropharyngeal HNSCC (49.1%).

**Conclusions:**

PLWH who receive care in the VA had higher age‐adjusted HNSCC incidence rates than reported in the general population, suggesting that HIV and immunosuppression play a role. Additional studies should be conducted to study the interaction between HPV and HIV.

## Graphical abstract

The elevated risk of both oropharynx cancer and non‐oropharyngeal head and neck cancer and poor survival among people living with HIV in the VA healthcare system suggests the host immune system interacts with smoking and HPV.

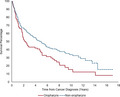

## 1 INTRODUCTION

In the United States, approximately, 1.1 million people are living with HIV.^1^ The total number of newly diagnosed HIV cases has remained stable between 2012 and 2016, with about 38,000 new cases a year.^1^ Since the introduction of effective combination antiretroviral therapy (ART), the life expectancy of people living with HIV/AIDS (PLWH) has been approaching that of the non‐HIV population.^2^ Thus, there is a concern of an increasing rate of non‐AIDS‐defining cancers, including head and neck cancers, whose incidence sharply increases with aging in both HIV‐infected and non‐infected populations. PLWH have a higher incidence of both virus‐related and tobacco/alcohol‐related cancers due to a higher prevalence of HIV‐induced inflammation, immunodeficiency, and tobacco use among PLWH compared with the non‐HIV population.^3^, ^4^ Indeed, as many as 20% of deaths among people living with HIV/AIDS is due to invasive non‐AIDS‐defining cancers.

Squamous cell carcinoma of the head and neck region (HNSCC) includes the oral cavity, oropharynx, hypopharynx, and larynx. Since HNSCC is strongly associated with tobacco use, the incidence for most of the sites has been decreasing.^5^, ^6^ In stark contrast, the incidence of squamous cell carcinoma of the oropharynx (OPSCC) is rising at an epidemic rate due to its relationship with the human papillomavirus (HPV);^6^ OPSCC has now overtaken cervical cancer as the most common HPV‐positive malignancy.^7^ Prior studies have shown that PLWH have an excess risk of HNSCC compared to the non‐HIV population, which could be due to a higher prevalence of tobacco and alcohol use. However, the elevated risk of lung cancer––another smoking‐related cancer––is not entirely explained by smoking, suggesting HIV‐related immune suppression and chronic inflammation are also likely contributors.^8^ The drivers of increased HNSCC risk in PLWH patients are entirely unclear in the context of HPV‐positive OPSCC. Since both HPV‐negative and HPV‐positive head and neck cancers have high levels of immune infiltrate,^9^ suggesting the immune system plays an essential role in HNSCC development and survival.

Given the heterogeneity in the risk of HNSCC in people living with HIV and the importance of the immune system, we aimed to identify risk factors associated with oropharyngeal and non‐oropharyngeal HNSCC in a large retrospective cohort study of PLWH identified in the U.S. national Veterans Health Administration (VHA) system. We also examined factors associated with survival among veterans living with HIV with HNSCC.

## 2 METHODS

### 2.1 Study population and design

We performed a retrospective cohort study using individual‐level patient data from the national VA Corporate Data Warehouse (CDW) and the VA Central Cancer Registry (CCR) from 1999 to 2015. Detailed information about cohort construction and inclusion has been previously described.^10^ Briefly, the cohort of PLWH included patients aged 18 or above who fulfilled two out of the three following criteria: (1) patients with at least one positive HIV antibody test by Elisa or Western Blot; tested for HIV viral load (any +/‐/indeterminate); or tested for CD4+ count; (2) patients with at least one prescription in inpatient or outpatient pharmacy records for HIV antiretroviral therapy (ART); and (3) any inpatient or outpatient encounter with an International Classification of Diseases, Ninth Revision (ICD‐9) (042 or V08) or ICD‐10 code (B20 & Z21) for HIV. We used the earliest date of the HIV diagnostic criteria as the index date of follow‐up unless it was before 10/01/1999 (inception date of CDW data), in which case we assigned 10/01/1999 as the HIV patient's index date.

### 2.2 Variable specification

#### 2.2.1 Outcomes

**Figure 1:**
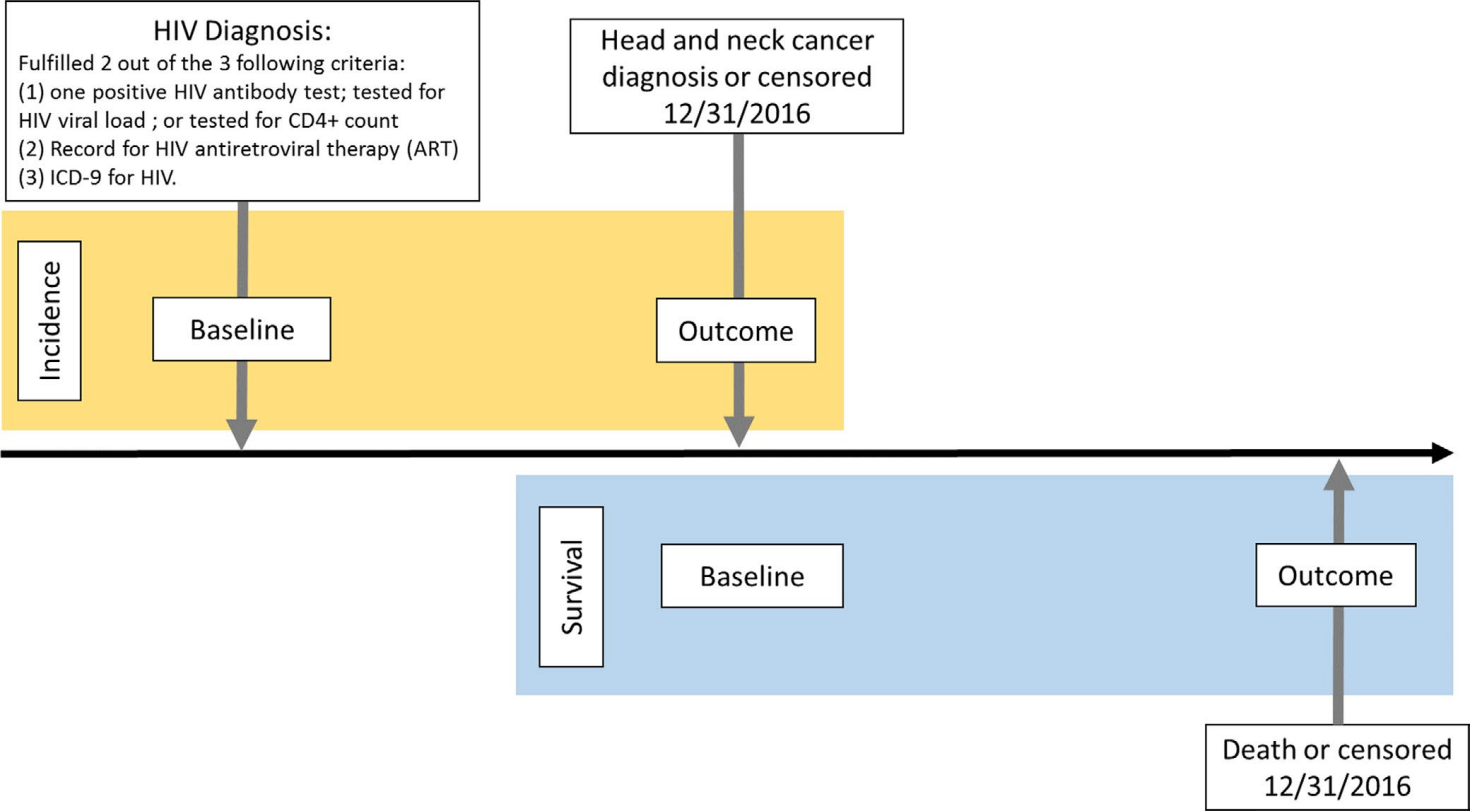
Schematic for the incidence (yellow box) and survival (blue box) study design

We are interested in two outcomes, first is HNSCC among the whole cohort and second survival among the HNSCC cohort (Figure 1). For the first incidence outcome (yellow box in Figure 1), we used a hierarchical approach to define the occurrence of OPSCC or non‐oropharyngeal HNSCC in the cohort. First, we identified all head and neck squamous cancer cases in the VA CCR based on primary site of OPSCC and non‐oropharyngeal HNSCC defined as oral cavity, hypopharynx, and larynx cancer. We then identified patients with any ICD‐9 or ICD‐10 code for HNSCC (Table S1). We examined the discordance of cancer diagnosis between the VA CCR and ICD‐9/ICD‐10 with a manual review of the electronic medical record (EMR) to determine their true cancer status. This hierarchical approach ensured a high validity of all the captured cancer cases. We then excluded all prevalent cancer cases that were diagnosed any time before the HIV index date through to 90 days after the index date.

For the second outcome of mortality (blue box in Figure 1), all‐cause mortality was identified from the VA Vital Status file, which combines information from Medicare, VA, Social Security, and VA compensation and pension benefits to determine the date of death with a sensitivity of 98.3% and specificity of 99.8% when compared with the National Death Index.^11^ During follow‐up, only one female developed non‐oropharyngeal HNSCC, and none developed OPSCC. Thus, we excluded female patients from this study.

#### 2.2.2 Covariates

We used a previously reported method to abstract and to classify covariates.^12^ Briefly, we extracted demographic data and risk factor information using ICD codes and pharmacy and laboratory data from the CDW, including age at index date, sex, race/ethnicity (White, Black, and other/unknown), history of alcohol abuse (yes, no), and smoking (ever or missing, never), and gastroesophageal reflux disease (GERD; ever, never). We defined baseline body mass index (BMI) by using the closest height and weight values in the 2 years before or after the index date. HIV‐specific variables included were nadir CD4 count (cells/μL) and percent of follow‐up time with undetectable HIV viral load, both as time‐updated variables throughout the study period.

### 2.3 Statistical analysis

#### 2.3.1 Incidence and risk factors for HNSCCs in veterans living with HIV

We compared baseline characteristics between patients with OPSCC, non‐oropharyngeal HNSCC, and patients without an HNSCC diagnosis (i.e., controls) using a student's t‐test for continuous variables and chi‐square tests for categorical variables. Therefore, follow‐up time at risk was calculated from 90 days after the index date––the possible earliest start date of follow‐up was 1/1/2000––to the development of HNSCC, death, or 12/31/2016, whichever was earlier. We calculated incidence rates age‐standardized to the 2000 U.S. population for HNSCC in the PLWH per 100,000 person‐years (PY) in oropharyngeal and non‐oropharyngeal HNSCC separately. We then estimated adjusted hazard ratios (HR) and 95% confidence intervals (CIs) for the risks of non‐oropharyngeal HNSCC and OPSCC using Cox proportional hazards regression models from index date plus 90 days to cancer or censor. We used forward selection to include covariates that changed estimates more than 10% or deemed clinically relevant. We adjusted for age at index, race, smoking, baseline BMI, GERD, viral load percentage of time undetectable (time‐dependent), nadir CD4 (time‐dependent), and year first HIV diagnosis.

#### 2.3.2 Risk factors for mortality among People Living with HIV with HNSCC

We also examined survival using Kaplan–Meier curves for both oropharyngeal and non‐oropharyngeal HNSCC as well as risk factors for mortality among PWLH with these cancers using Cox proportional hazards regression analysis the cancer date to death or censor. We examined the same potential confounder variables as examined in our Cox models for cancer incidence, as described above.

#### 2.3.3 HPV status exploratory analysis

To estimate the effect of HPV in OPSCC, we abstracted HPV status from medical records for all OPSCCs diagnosed after 2010 (HPV testing was not recommended 2010).^13^ Cases were classified as HPV‐positive if the case was positive for either p16 immunohistochemistry or HPV *in situ* hybridization and as HPV‐negative if one test was negative and the other was unknown or untested. We compared univariate demographics and survival between HPV‐positive, HPV‐negative, and unknown HPV status at OPSCC diagnosis using statistics described above. We did not perform Cox regression given limited power due to reduced sample size.

Analyses were performed using SAS version SAS Enterprise Guide Version 7.15 (SAS Institute, Cary, NC). Statistical significance was determined at α=0.05, and P‐values for statistical significance were two‐sided.

## 3 RESULTS

### 3.1 Descriptive statistics

We included data from 44,052 veterans living with HIV/AIDS using VA healthcare. (Table 1). As expected, in our cohort, patients with OPSCC were younger than patients with non‐oropharyngeal HNSCC (11.8% vs 17.8% older than 60 years at HIV index date, respectively) and controls (12.4% older than 60 years at HIV index date). OPSCC cases were more likely to be lifelong non‐smokers compared with non‐oropharyngeal HNSCC cases (12.6% vs 8.1% non‐smoker, respectively) and controls (19.7%). OPSCC cases were more likely to have a history of alcohol abuse (54.6% in OPSCC vs 41.9% non‐oropharyngeal HNSCC) and substance abuse (53.8% in OPSCC vs 40.4% non‐oropharyngeal HNSCC) compared with both noncancer subjects and non‐oropharyngeal HNSCC cases.

**Table 1:** Descriptive statistics of PLWH who received healthcare at VA from 1999 to 2015 by head and neck cancer diagnosis.

	No cancer	Oropharyngeal	Non‐oropharyngeal	*p*‐value
	n = 43,663	n = 119	n = 270	
	n (%)	n (%)	n (%)	
Age at index date				
<40	9889 (22.7)	14 (11.8)	28 (10.4)	<0.001
40‐59	28350 (64.9)	91 (76.5)	194 (71.9)	
60 and above	5424 (12.4)	14 (11.8)	48 (17.8)	
Race				
Black	22847 (52.3)	68 (57.1)	131 (48.5)	0.041
Other/Unknown	4257 (9.8)	6 (5.0)	18 (6.7)	
White	16559 (37.9)	45 (37.8)	121 (44.8)	
Smoking				
Ever or unknown smoker	35079 (80.3)	104 (87.4)	248 (91.9)	<0.001
Lifelong non‐smoker	8584 (19.7)	15 (12.6)	22 (8.1)	
Alcohol abuse				
No	24605 (56.4)	54 (45.4)	157 (58.1)	0.046
Yes	19058 (43.6)	65 (54.6)	113 (41.9)	
Substance abuse				
No	24957 (57.2)	55 (46.2)	161 (59.6)	0.039
Yes	18706 (42.8)	64 (53.8)	109 (40.4)	
Baseline BMI				
Missing	1543 (3.5)	3 (2.5)	7 (2.6)	<0.001
<25	21063 (48.2)	77 (64.7)	157 (58.2)	
25‐29.9	14559 (33.3)	26 (21.8)	84 (31.1)	
>30	6498 (14.9)	13 (10.9)	22 (8.1)	
GERD (ever)				
No	33277 (76.2)	78 (65.6)	166 (61.5)	<0.001
Yes	10386 (23.8)	41 (34.5)	104 (38.5)	
HCV (ever)				
No	33882 (77.6)	80 (67.2)	198 (73.3)	0.006
Yes	9781 (22.4)	39 (32.8)	72 (26.7)	
Nadir CD4 (at cancer/censor)				
Missing	1409 (3.2)	2 (1.7)	10 (3.7)	0.061
≤200	21159 (48.5)	63 (52.9)	152 (56.3)	
>200	21095 (48.3)	54 (45.4)	108 (40.0)	
Viral load percentage of time undetectable (at cancer/censor)				
Missing	1175 (2.7)	1 (0.8)	3 (1.1)	0.002
<40%	11081 (25.4)	34 (28.6)	98 (36.3)	
40%‐80%	11933 (27.3)	35 (29.4)	66 (24.4)	
>80%	19474 (44.6)	49 (41.2)	103 (38.2)	
Year first HIV				
Before 1996	8582 (19.7)	41 (34.5)	80 (29.6)	<0.001
1996‐2000	10161 (23.3)	38 (31.9)	93 (34.4)	
2001‐2005	9240 (21.2)	20 (16.8)	63 (23.3)	
2006‐2016	15680 (35.9)	20 (16.8)	34 (12.6)	

Abbreviations: BMI, Body Mass Index; GERD, Gastroesophageal reflux disease; HCV, Hepatitis C virus; PLWH, People living with HIV.

### 3.2 Incidence of OPSCC and non‐oropharyngeal HNSCC in PLWH

Of the 44,052 PLWH in our nationwide VA cohort, there were 119 cases of incident OPSCC and 270 cases of non‐oropharyngeal HNSCC. The age‐standardized rate of OPSCC was 23.0 (95% CI: 17.1‐28.9) per 100,000 PY. The age‐standardized rate of non‐oropharyngeal HNSCC was higher at 55.4 (95% CI: 46.5‐64.3) per 100,000 PY.

In the adjusted Cox regression (Table 2), age greater than 60 years was associated with increased risk of both OPSCC (HR: 3.35; 95% CI: 1.56‐7.18) and non‐oropharyngeal HNSCC (HR: 5.80; 95% CI: 3.58‐9.39) compared with age <40 years in PLWH. Ever smoking was only associated with risk of developing non‐oropharyngeal HNSCC but not OPSCC (HR: 2.38; 95% CI: 1.53‐3.71 vs HR: 1.28; 95% CI: 0.73‐2.23, respectively). Nadir CD4 ≤200 was associated with an increased risk of non‐oropharyngeal HNSCC (HR: 1.78; 95% CI: 1.31‐2.30). GERD was also associated with the risk of non‐oropharyngeal HNSCC (HR: 1.46; 95% CI: 1.14‐1.87). Year of HIV diagnosis was significantly associated with decreased risk of both OPSCC (HR: 0.49; 95% CI: 0.28‐0.85 for 2001‐2005 vs prior to 1999) and non‐oropharyngeal HNSCC (HR: 0.56; 95% CI: 0.36‐0.88 for 2006‐2016 vs prior to 1999).

**Table 2:** Mutually adjusted hazard ratios for the risk of oropharyngeal and non‐oropharyngeal cancer in PLWH receiving healthcare at VA from 1999 to 2015.

	Oropharyngeal		Non‐oropharyngeal	
	HR (95% CI)	*p*‐value	HR (95% CI)	*p*‐value
Age at index				
<40	1.00		1.00	
40‐59	2.17 (1.23, 3.84)	0.008	2.35 (1.57, 3.50)	<0.001
60 and above	3.35 (1.56, 7.18)	0.002	5.80 (3.58, 9.39)	<0.001
Race				
White	1.000		1.00	
Black	1.00 (0.68, 1.47)	0.993	0.77 (0.59, 0.99)	0.040
Other/Unknown	0.65 (0.28, 1.54)	0.329	0.75 (0.46, 1.24)	0.264
Smoking				
Lifelong non‐smoker	1.00		1.00	
Ever or unknown smoker	1.28 (0.73, 2.23)	0.386	2.38 (1.53, 3.71)	<0.001
Alcohol				
No	1.00		1.00	
Yes	1.81 (1.23, 2.66)	0.003	1.16 (0.90, 1.51)	0.253
Baseline BMI				
<25	1.00		1.00	
25‐29.9	0.48 (0.31, 0.76)	0.001	0.79 (0.60, 1.03)	0.081
>30	0.63 (0.35, 1.14)	0.130	0.56 (0.35, 0.87)	0.010
Missing	0.50 (0.16, 1.62)	0.250	0.52 (0.24, 1.12)	0.092
GERD				
No	‐‐		1.00	
Yes	‐‐	‐‐	1.46 (1.14, 1.87)	0.003
Viral load percentage of time undetectable (time‐dependent)				
<40%	1.00		1.00	
40%‐80%	1.19 (0.73, 1.95)	0.486	0.78 (0.56, 1.09)	0.144
>80%	1.29 (0.81, 2.07)	0.283	0.85 (0.63, 1.15)	0.288
Missing	1.50 (0.18, 12.28)	0.708	0.92 (0.27, 3.16)	0.895
Nadir CD4 (time‐dependent)				
>200	1.00		1.00	
≤200	1.39 (0.95, 2.02)	0.088	1.78 (1.31, 2.30)	<0.001
Missing	0.38 (0.51, 2.28)	0.112	1.14 (0.56, 2.30)	0.721
Year first HIV				
Before 1996	1.00		1.00	
1996‐2000	0.82 (0.52, 1.27)	0.366	1.02 (0.76, 1.38)	0.880
2001‐2005	0.49 (0.28, 0.85)	0.011	0.83 (0.59, 1.17)	0.282
2006‐2016	0.69 (0.38, 1.26)	0.223	0.56 (0.36, 0.88)	0.011

Abbreviations: BMI, Body Mass Index; GERD, Gastroesophageal reflux disease; PLWH, People living with HIV.

### 3.3 Survival for HNSCC in PLWH patients

**Figure 2:**
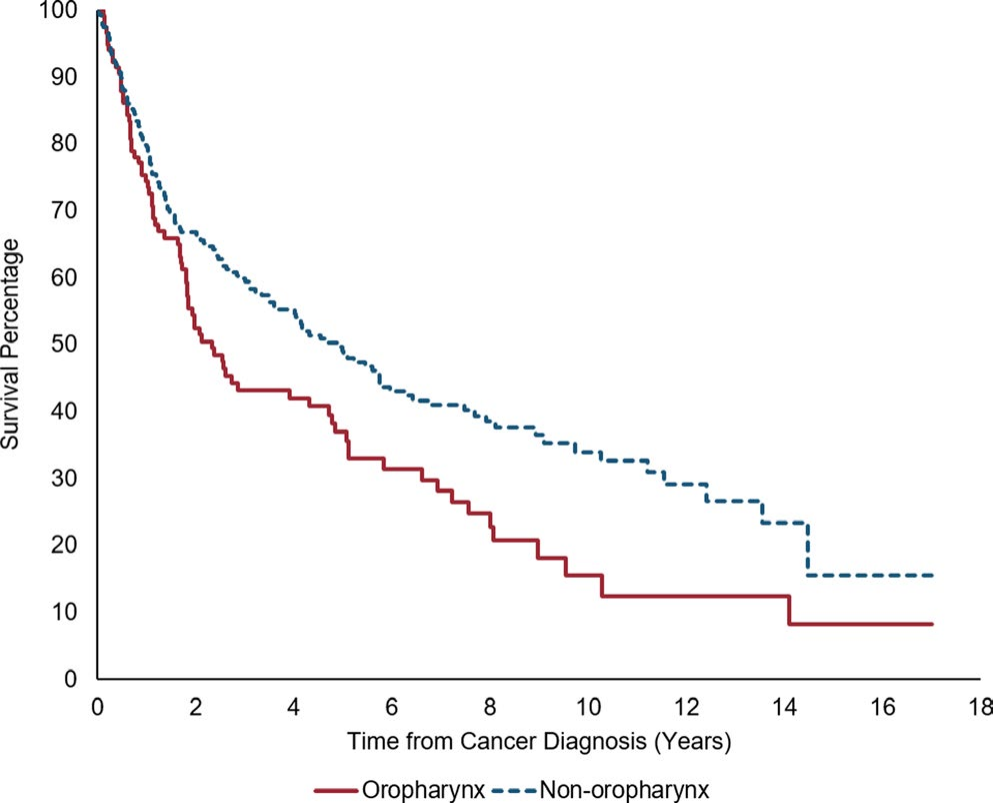
Kaplan–Meier curve for survival after between oropharyngeal and non‐oropharyngeal cancer

Overall survival (OS) for OPSCC (5‐year OS: 37.0%; Figure 2) was significantly lower than that of non‐oropharyngeal HNSCC (5‐year OS: 49.1%; log‐rank p‐value=0.005). We further categorized non‐oropharyngeal HNSCC into hypopharynx (n = 24), larynx (n = 120), nasopharynx (n = 11) and oral cavity (n = 115). Five‐year OPSCC OS was lower than survival for both larynx (45.6%) and oral cavity cancer (65.4%; Figure S1A). These trends also continued when stratified by smoking, where smokers with OPSCC had a worse survival than smokers with non‐oropharyngeal HNSCC (Figure S1B). The 5‐year OS in stage III–IV was lower than stage I‐II in both OPSCC (I–II: 40.4%, III–IV: 30.9%, Figure S1C) and non‐oropharyngeal HNSCC (I–II: 66.2%, III–IV: 22.67%, Figure S1D).

In multivariable Cox analysis for survival, ever smoking was significantly associated with increased risk of death, and BMI was associated with a decreased risk of death among both OPSCC and non‐oropharyngeal HNSCC patients (Table 3). Although not significant, nadir CD4 count was also associated with OPSCC survival, where cases with nadir CD4 count less than or equal to 200 had 2.00 times the hazard of death (95% CI: 0.94‐4.27) compared to those with nadir CD4 greater than 200.

**Table 3:** Mutually adjusted hazard ratios for risk of mortality among PLWH in the VA after oropharyngeal and non‐oropharyngeal cancer diagnosis.

	Oropharynx (N event =80)			Non‐oropharynx (N event =147)		
	N (%)	HR (95% CI)	*p*‐value	N (%)	HR (95% CI)	*p*‐value
Age at index date						
<40	14 (11.8)	1.00		28 (10.4)	1.00	
40‐59	91 (76.5)	1.04 (0.40, 2.69)	0.939	194 (71.9)	5.91 (1.85, 18.87)	0.003
60 and above	14 (1.8)	2.41 (0.75, 7.80)	0.141	48 (17.8)	9.04 (2.68, 30.45)	<0.001
Smoking						
Non‐smoker	104 (87.4)	1.00		248 (91.9)	1.00	
Ever or unknown smoker	15 (12.6)	2.96 (1.25, 6.99)	0.013	22 (8.1)	3.31 (1.35, 8.12)	0.009
Baseline BMI						
<25	77 (66.4)	1.00		157 (59.5)	1.00	
25‐29.9	26 (22.4)	0.80 (0.43, 1.49)	0.489	85 (32.2)	0.51 (0.34, 0.76)	0.001
>30	13 (11.2)	0.26 (0.09, 0.74)	0.012	22 (8.3)	0.86 (0.41, 1.81)	0.690
Viral load percentage of time undetectable (time‐dependent*)						
<40%	28 (23.7)	1.00		61 (22.8)	1.00	
40%‐80%	39 (33.1)	1.31 (0.73, 2.34)	0.369	98 (36.7)	0.89 (0.58, 1.36)	0.588
>80%	51 (43.2)	0.71 (0.38, 1.32)	0.284	108 (40.4)	0.78 (0.51, 1.19)	0.244
Nadir CD4 (time‐dependent*)						
>200	106 (89.1)	1.00		195 (73.0)	1.00	
≤200	13 (10.9)	2.00 (0.94, 4.27)	0.072	72 (27.0)	1.11 (0.75, 1.64)	0.597
Stage						
I‐II	22 (18.5)	1.00		119 (44.1)	1.00	
III‐IV	86 (72.3)	1.52 (0.86, 2.71)	0.153	94 (34.8)	2.63 (1.79, 3.88)	<0.001
Unknown	11 (9.2)	0.70 (0.22, 2.26)	0.549	57 (21.1)	1.55 (0.97, 2.48)	0.069

Abbreviation: BMI, body mass index.

* N (%) is at cancer/censor

### 3.4 HPV impact on OPSCC survival in PLWH

**Figure 3:**
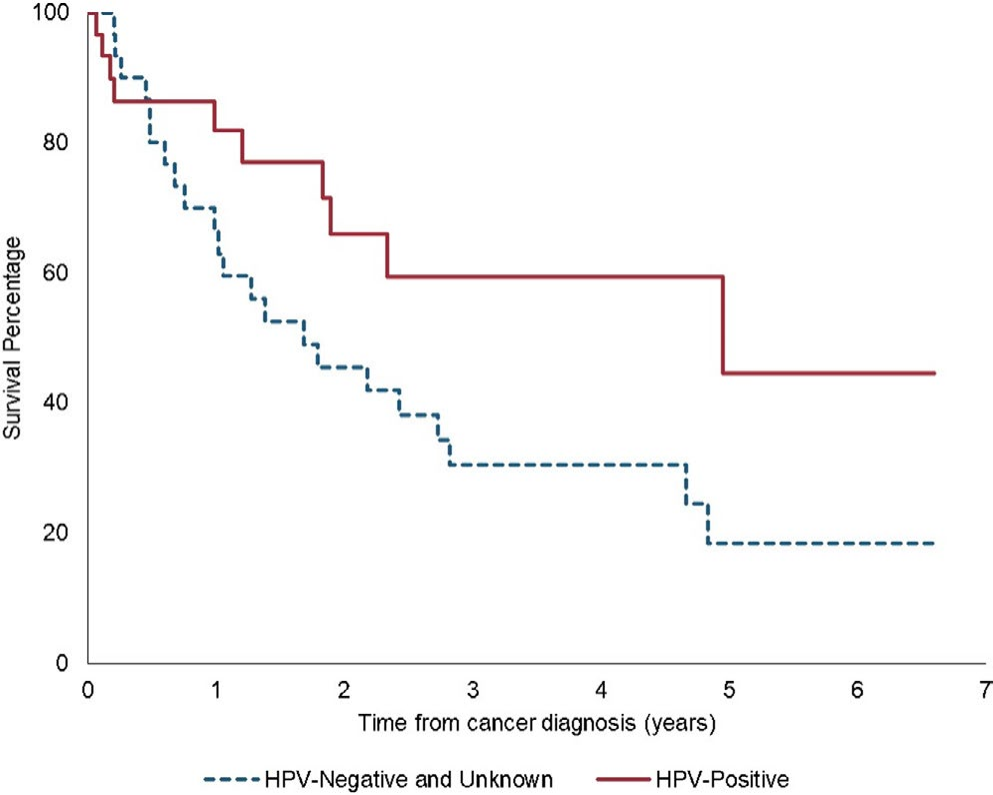
Survival among PLWH in the VA with oropharyngeal cancer by p16/HPV status

In our sensitivity analysis examining HPV in OPSCC, we found of the 62 cases diagnosed since 2010, 56.4% (n = 35) were tested for HPV. Of these, 88.6% (n = 31) were HPV‐positive and 11.4% (n = 4) were HPV‐negative. Older individuals, smokers, and those whose cancer was first diagnosed before 2013 were less likely to be tested (Table S2). In a Kaplan–Meier curve (Figure 3), HPV‐negative and unknown cases had worse OS (5‐year OS: 18.3%) than HPV‐positive cases (5‐year OS: 59.4%).

## 4 DISCUSSION

In this large retrospective cohort study of veterans living with HIV using the nationwide VA healthcare system, we found that the age‐standardized rate of non‐oropharyngeal HNSCC is higher compared with age‐standardized rates of OPSCC. The HIV‐specific variable associated with non‐oropharyngeal HNSCC was nadir CD4 count, which was associated with an increased risk of cancer. We are also the first study to demonstrate lower survival in OPSCC compared with laryngeal and oral cavity cancer in both PLWH and veterans. This trend is consistent when stratified by stage. Upon further inspection, we found evidence that, although HPV‐positive OPSCC has better survival than non‐HPV OPSCC, survival is still lower in PLWH then the non‐HIV population.

The incidence of HNSCC is higher in PWLH compared with the general SEER population,^14^, ^15^ which is paralleled in our study where we see a higher incidence of both OPSCC and non‐oropharyngeal HNSCC. PLWH tend to have more risk factors for cancer, such as risky sexual behavior, smoking, and immunosuppression.^16^ The age‐standardized incidence of OPSCC and non‐oropharyngeal HNSCC in our study is higher than previously reported in North American AIDS Cohort Collaboration on Research and Design (NA‐ACCORD) consortium (40 per 100,000 person‐years and 10.7 per 100,000 person‐years, respectively).^3^ The NA ACCORD consortium has pooled data from 17 prospective studies, including data from the VHA. However, our cohort was developed separately from the Veterans Aging Cohort (VACS) using a separate methodology. Additionally, male veterans who have a higher prevalence of cancer risk factors such as smoking and alcohol use thus represents a different target population from NA ACCORD.^17^

Overall, we see results similar to those observed in the NA ACCORD consortium. Age appears to one of the most significant risk factors for HNSCC among PLWH, which is relevant given the aging PLWH population. We found no association between HIV‐related factors and OPSCC, supporting previous research that immunosuppression is more relevant for non‐HPV associated HNSCC than HPV‐positive cancer.^18^, ^19^ The increased but nonsignificant association of CD4 count may be due to the impact of CD4 early in HPV‐positive cancer by affecting HPV persistence. ^20^, ^21^

On the contrary, for non‐HPV‐positive cancer, CD4 count would reflect reduced immune surveillance for malignant cells in the development of HNSCC.^22^ Alternatively, there is evidence that CD4 count decline could be due to undiagnosed cancer, rather than causing cancer. However, this has mainly been observed in lymphomas, and, in our study, the nadir CD4 count associated with cancer could be many years before the incident cancer diagnosis.

The relationship between GERD and non‐oropharyngeal HNSCC is mixed with many earlier studies suggesting an association with hypopharynx or larynx cancer, but recent studies have not supported these findings.^23^, ^24^, ^25^, ^26^ Our study is the first study of PLWH to examine GERD, in which we observed an association of GERD with non‐oropharyngeal HNSCC. However, this association could be due to reverse causality, where the symptoms of laryngeal cancer mimic those of GERD. Thus, both are more likely diagnosed. Future studies may determine if this increased risk is simply due to detection bias.

This is the first large cohort study of HNSCC among PLWH that includes survival and stage. Survival in this cohort is lower than the SEER population for both OPSCC and non‐oropharyngeal HNSCC. The lower survival compared to the non‐veteran and non‐HIV population could be due to the increased prevalence of smoking and medical conditions in the VA in addition to decreased immune response due to HIV. Low CD4 count is associated with increased risk of death in OPSCC, but not non‐oropharyngeal HNSCC. HPV‐positive OPSCC tumors tend to have more immune infiltrated and have higher levels of T cells. We also found the survival of OPSCC to be much lower than the survival of non‐oropharyngeal HNSCC, even given the lower prevalence of suggesting there is an interaction between HIV and HPV in OPSCC. Coupled with increased risk among PLWH, immunity may play a more crucial role in HPV‐positive cancers than in non‐HPV‐positive HNSCCs.

In a subset analysis, we found that among the cases tested for HPV, the majority are HPV‐positive, which is similar to previous studies of HPV status in OPSCC in the VA.^27^ We also found that although HPV‐positive OPSCC cases have better survival than HPV‐negative OPSCC, they still have lower survival than the non‐HIV population. Two‐year survival for HPV‐positive OPSCC for all stages has been reported to be as high at 90% in the non‐HIV non‐Veteran population.^28^, ^29^ This difference in survival signifies that host immune likely plays an essential role in the increased survival of HPV‐positive OPSCC. Smoking is also a likely driver of poor outcomes, particularly in the Veteran population with high rates of tobacco use. Smoking worsens prognosis in HPV‐positive OPSCC, increasing the risk of death by 1% for every one pack‐year of smoking.^30^, ^31^, ^32^ However, to date, few studies have considered the interaction between HIV and HPV in OPSCC, and one previous study of OPSCC among PWLH found no difference in survival by HPV status,^33^ This discordance justifies additional examination of the interaction between HPV and HIV in OPSCC specifically.

Given both the increased risk of HNSCC and higher rates of death from HNSCC among PLWH, there is a strong case for dysfunctional immunity as a driver of poor HNSCC outcomes in this population. Both HPV‐ and tobacco‐associated HNSCCs have an immunosuppressive tumor immune microenvironment.^34^, ^35^ Specifically, HPV‐positive OPSCC inherently represents a failure of host immunity, allowing HPV infection, persistence, and progression to carcinoma go unchecked. In the non‐HIV population, improved survival may be in part due to enhanced immune response induced by radiation therapy.^9^, ^36^ ART‐associated immune reconstitution fails to completely restore immune populations required for successful control of cancer after radiation therapy.

Our study has several limitations. First, it was a historical cohort and may be subject to unmeasured confounders such as sexual behavior and misclassification of covariates. However, we attempted to adjust for this through ICD‐9 codes identifying individuals with a history of both alcohol and drug use. Although we were able to abstract HPV data on some of the OPSCC cases, we lacked information about the tumor HPV status of for most of the OPSCC cases, since only about half the cases after 2010 were tested for HPV. Finally, this study was limited to male veterans. Veterans are a select group that has lower socioeconomic status, more medical conditions, which would limit the generalizability to the other populations of PWLH.^37^

Our study has multiple notable strengths. The VHA is the most extensive integrated healthcare system and provider of comprehensive HIV care in the United States. By taking advantage of the comprehensive fully automated nationwide VA clinical, laboratory, and administrative databases (CDW and CCR) along with manual electronic medical records abstractions, we constructed a large cohort of PLWH with extensive follow‐up and individual‐level data. Additionally, this is the largest study published to date evaluating HNSCC survival among PLWH.

In summary, HNSCC is more common among PLWH than in the general U.S. population. Certain HIV‐specific variables such as CD4 count are associated with increased risk suggesting that HIV, and thus, the host immune system, in addition to increased prevalence of head and neck risk factors such as smoking and HPV, increases the risk of HNSCC. As the prevalence of both HIV rises in the United States due to better survival and the incidence of HPV‐positive OPSCC increases, the interaction between the HPV and HIV becomes increasingly relevant, especially since HPV‐positive OPSCCs among PLWH in our cohort do not have the generally favorable survival seen in the non‐HIV population. This growing concern needs a dedicated effort and resources as well as additional studies to study the interaction between HPV, tobacco, and HIV in OPSCC.

## AUTHOR CONTRIBUTIONS

EC, JK, and DLW conceived and designed this study. STA, KR, CH, and SR collected and curated the data. CH performed the data analysis. ALM, CH, KR, DLM, PR, and EC were involved with the interpretation. ALM drafted the article. ALM, CH, KR, DLW, KR, SR, VS, STA, PR, AGS, and EC provided critical revision. All authors approved of the version to be published.

## Supporting information

Supplementary Material

## Data Availability

Data subject to third party restrictions. The data that support the findings of this study are available from Veteran Affairs. Restrictions apply to the availability of these data, which were used under license for this study. Data are available from the authors with the permission of Veteran Affairs.
