# Supplementary material for: Incidence and survival for oropharynx and non‐oropharynx head and neck cancers among veterans living with HIV
Source: Cancer Med. 2020 Oct 23;9(24):9326–35. doi: 10.1002/cam4.3539 (PMC7774719; doi:10.1002/cam4.3539)

**Supplemental table 1**. ICD-9 and ICD-10 codes for head and neck cancer

| **ICD-9** | |  |  | **ICD-10** |
| --- | --- | --- | --- | --- |
| **ICD-9-CM CODE** | **ICD-9-CM CODE DESCRIPTION** |  | **ICD-10-CM CODE** | **ICD-10-CM CODE DESCRIPTION** |
| 1400 | Mal Neo Upper Vermilion |  | C000 | Malignant neoplasm of external upper lip |
| 1401 | Mal Neo Lower Vermilion |  | C001 | Malignant neoplasm of external lower lip |
| 1403 | Mal Neo Upper Lip- Inner |  | C002 | Malignant neoplasm of external lip, unspecified |
| 1404 | Mal Neo Lower Lip- Inner |  | C003 | Malignant neoplasm of upper lip, inner aspect |
| 1405 | Mal Neo Lip- Inner NOS |  | C004 | Malignant neoplasm of lower lip, inner aspect |
| 1406 | Mal Neo Lip- Commissure |  | C005 | Malignant neoplasm of lip, unspecified, inner aspect |
| 1408 | Mal Neo Lip NEC |  | C006 | Malignant neoplasm of commissure of lip, unspecified |
| 1409 | Mal Neo Lip/Vermil NOS |  | C008 | Malignant neoplasm of overlapping sites of lip |
| 1410 | Mal Neo Tongue Base |  | C009 | Malignant neoplasm of lip, unspecified |
| 1411 | Mal Neo Dorsal Tongue |  | C01 | Malignant neoplasm of base of tongue |
| 1412 | Mal Neo Tip/Lat Tongue |  | C020 | Malignant neoplasm of dorsal surface of tongue |
| 1413 | Mal Neo Ventral Tongue |  | C021 | Malignant neoplasm of border of tongue |
| 1414 | Mal Neo Ant 2/3 Tongue |  | C022 | Malignant neoplasm of ventral surface of tongue |
| 1415 | Mal Neo Tongue Junction |  | C023 | Malig neoplasm of anterior two-thirds of tongue, part unsp |
| 1416 | Mal Neo Lingual Tonsil |  | C024 | Malignant neoplasm of lingual tonsil |
| 1418 | Malig Neo Tongue NEC |  | C028 | Malignant neoplasm of overlapping sites of tongue |
| 1419 | Malig Neo Tongue NOS |  | C029 | Malignant neoplasm of tongue, unspecified |
| 1420 | Malig Neo Parotid |  | C030 | Malignant neoplasm of upper gum |
| 1421 | Malig Neo Submandibular |  | C031 | Malignant neoplasm of lower gum |
| 1422 | Malig Neo Sublingual |  | C039 | Malignant neoplasm of gum, unspecified |
| 1428 | Mal Neo Maj Salivary NEC |  | C040 | Malignant neoplasm of anterior floor of mouth |
| 1429 | Mal Neo Salivary NOS |  | C041 | Malignant neoplasm of lateral floor of mouth |
| 1430 | Malig Neo Upper Gum |  | C048 | Malignant neoplasm of overlapping sites of floor of mouth |
| 1431 | Malig Neo Lower Gum |  | C049 | Malignant neoplasm of floor of mouth, unspecified |
| 1438 | Malig Neo Gum NEC |  | C050 | Malignant neoplasm of hard palate |
| 1439 | Malig Neo Gum NOS |  | C051 | Malignant neoplasm of soft palate |
| 1440 | Mal Neo Ant Floor Mouth |  | C052 | Malignant neoplasm of uvula |
| 1441 | Mal Neo Lat Floor Mouth |  | C058 | Malignant neoplasm of overlapping sites of palate |
| 1448 | Mal Neo Mouth Floor NEC |  | C059 | Malignant neoplasm of palate, unspecified |
| 1449 | Mal Neo Mouth Floor NOS |  | C060 | Malignant neoplasm of cheek mucosa |
| 1450 | Mal Neo Cheek Mucosa |  | C061 | Malignant neoplasm of vestibule of mouth |
| 1451 | Mal Neo Mouth Vestibule |  | C062 | Malignant neoplasm of retromolar area |
| 1452 | Malig Neo Hard Palate |  | C0680 | Malignant neoplasm of ovrlp sites of unsp parts of mouth |
| 1453 | Malig Neo Soft Palate |  | C0689 | Malignant neoplasm of overlapping sites of oth prt mouth |
| 1454 | Malignant Neoplasm Uvula |  | C069 | Malignant neoplasm of mouth, unspecified |
| 1455 | Malignant Neo Palate NOS |  | C07 | Malignant neoplasm of parotid gland |
| 1456 | Malig Neo Retromolar |  | C080 | Malignant neoplasm of submandibular gland |
| 1458 | Malig Neoplasm Mouth NEC |  | C081 | Malignant neoplasm of sublingual gland |
| 1459 | Malig Neoplasm Mouth NOS |  | C089 | Malignant neoplasm of major salivary gland, unspecified |
| 1460 | Malignant Neopl Tonsil |  | C090 | Malignant neoplasm of tonsillar fossa |
| 1461 | Mal Neo Tonsillar Fossa |  | C091 | Malig neoplasm of tonsillar pillar (anterior) (posterior) |
| 1462 | Mal Neo Tonsil Pillars |  | C098 | Malignant neoplasm of overlapping sites of tonsil |
| 1463 | Malign Neopl Vallecula |  | C099 | Malignant neoplasm of tonsil, unspecified |
| 1464 | Mal Neo Ant Epiglottis |  | C100 | Malignant neoplasm of vallecula |
| 1465 | Mal Neo Epiglottis Junct |  | C101 | Malignant neoplasm of anterior surface of epiglottis |
| 1466 | Mal Neo Lat Oropharynx |  | C102 | Malignant neoplasm of lateral wall of oropharynx |
| 1467 | Mal Neo Post Oropharynx |  | C103 | Malignant neoplasm of posterior wall of oropharynx |
| 1468 | Mal Neo Oropharynx NEC |  | C104 | Malignant neoplasm of branchial cleft |
| 1469 | Malig Neo Oropharynx NOS |  | C108 | Malignant neoplasm of overlapping sites of oropharynx |
| 1470 | Mal Neo Super Nasopharyn |  | C109 | Malignant neoplasm of oropharynx, unspecified |
| 1471 | Mal Neo Post Nasopharynx |  | C110 | Malignant neoplasm of superior wall of nasopharynx |
| 1472 | Mal Neo Lat Nasopharynx |  | C111 | Malignant neoplasm of posterior wall of nasopharynx |
| 1473 | Mal Neo Ant Nasopharynx |  | C112 | Malignant neoplasm of lateral wall of nasopharynx |
| 1478 | Mal Neo Nasopharynx NEC |  | C113 | Malignant neoplasm of anterior wall of nasopharynx |
| 1479 | Mal Neo Nasopharynx NOS |  | C118 | Malignant neoplasm of overlapping sites of nasopharynx |
| 1480 | Mal Neo Postcricoid |  | C119 | Malignant neoplasm of nasopharynx, unspecified |
| 1481 | Mal Neo Pyriform Sinus |  | C12 | Malignant neoplasm of pyriform sinus |
| 1482 | Mal Neo Aryepiglott Fold |  | C130 | Malignant neoplasm of postcricoid region |
| 1483 | Mal Neo Post Hypopharynx |  | C131 | Malig neoplasm of aryepiglottic fold, hypopharyngeal aspect |
| 1488 | Mal Neo Hypopharynx NEC |  | C132 | Malignant neoplasm of posterior wall of hypopharynx |
| 1489 | Mal Neo Hypopharynx NOS |  | C138 | Malignant neoplasm of overlapping sites of hypopharynx |
| 1490 | Mal Neo Pharynx NOS |  | C139 | Malignant neoplasm of hypopharynx, unspecified |
| 1491 | Mal Neo Waldeyer-S Ring |  | C140 | Malignant neoplasm of pharynx, unspecified |
| 1498 | Mal Neo Oral/Pharynx NEC |  | C142 | Malignant neoplasm of Waldeyers ring |
| 1499 | Mal Neo Orophryn Ill-Def |  | C148 | Malig neoplm of ovrlp sites of lip, oral cavity and pharynx |
| 1600 | Mal Neo Nasal Cavities |  | C300 | Malignant neoplasm of nasal cavity |
| 1601 | Malig Neo Middle Ear |  | C301 | Malignant neoplasm of middle ear |
| 1602 | Mal Neo Maxillary Sinus |  | C310 | Malignant neoplasm of maxillary sinus |
| 1603 | Mal Neo Ethmoidal Sinus |  | C311 | Malignant neoplasm of ethmoidal sinus |
| 1604 | Malig Neo Frontal Sinus |  | C312 | Malignant neoplasm of frontal sinus |
| 1605 | Mal Neo Sphenoid Sinus |  | C313 | Malignant neoplasm of sphenoid sinus |
| 1608 | Mal Neo Access Sinus NEC |  | C318 | Malignant neoplasm of overlapping sites of accessory sinuses |
| 1609 | Mal Neo Access Sinus NOS |  | C319 | Malignant neoplasm of accessory sinus, unspecified |
| 1610 | Malignant Neo Glottis |  | C320 | Malignant neoplasm of glottis |
| 1611 | Malig Neo Supraglottis |  | C321 | Malignant neoplasm of supraglottis |
| 1612 | Malig Neo Subglottis |  | C322 | Malignant neoplasm of subglottis |
| 1613 | Mal Neo Cartilage Larynx |  | C323 | Malignant neoplasm of laryngeal cartilage |
| 1618 | Malignant Neo Larynx NEC |  | C328 | Malignant neoplasm of overlapping sites of larynx |
| 1619 | Malignant Neo Larynx NOS |  | C329 | Malignant neoplasm of larynx, unspecified |
| 1950 | Mal Neo Head/Face/NECk |  | C760 | Malignant neoplasm of head, face and NECk |
| 2300 | Ca In Situ Oral Cav/Phar |  | D0000 | Carcinoma in situ of oral cavity, unspecified site |
| 2310 | Ca In Situ Larynx |  | D0001 | Carcinoma in situ of labial mucosa and vermilion border |
| V1001 | Hx Of Tongue Malignancy |  | D0002 | Carcinoma in situ of buccal mucosa |
| V1002 | Hx-Oral/Pharynx Malg NEC |  | D0003 | Carcinoma in situ of gingiva and edentulous alveolar ridge |
| V1021 | Hx-Laryngeal Malignancy |  | D0004 | Carcinoma in situ of soft palate |
|  | |  | D0005 | Carcinoma in situ of hard palate |
|  |  |  | D0006 | Carcinoma in situ of floor of mouth |
|  |  |  | D0007 | Carcinoma in situ of tongue |
|  |  |  | D0008 | Carcinoma in situ of pharynx |
|  |  |  | D020 | Carcinoma in situ of larynx |
|  |  |  | Z8521 | Personal history of malignant neoplasm of larynx |
|  |  |  | Z85810 | Personal history of malignant neoplasm of tongue |
|  |  |  | Z85818 | Prsnl hx of malig neoplm of site of lip, oral cav, & pharynx |
|  |  |  | Z85819 | Prsnl hx of malig neoplm of unsp site lip,oral cav,& pharynx |

**Supplemental Table 2.** Descriptive statistics of PLWH in the VA with oropharyngeal cancer from 2010-2016 by HPV status.

|  | HPV-positive  n = 31 | HPV-negative  n = 4 | Not tested  n = 27 |  |
| --- | --- | --- | --- | --- |
|  | n (%) | n (%) | n (%) | p-value |
| Age at index date |  |  |  |  |
| < 40 | 8 (25.8%) | 0 (0.0%) | 2 (7.4%) | 0.1182 |
| 40-59 | 21 (67.7%) | 3 (50.0%) | 18 (66.7%) |  |
| 60 and above | 2 (6.5%) | 3 (50.0%) | 7 (25.9%) |  |
| Race |  |  |  |  |
| Black | 15 (48.4%) | 4 (100.0%) | 15 (55.6%) | 0.2379 |
| Other/Unknown | 2 (6.5%) | 0 (0.0%) | 0 (0.0%) |  |
| White | 14 (45.2%) | 0 (0.0%) | 12 (44.4%) |  |
| Smoking |  |  |  |  |
| Lifelong non-smoker | 7 (22.6%) | 0 (0.0%) | 1 (3.7%) | 0.0739 |
| Ever or unknown smoker | 24 (77.4%) | 4 (100.0%) | 26 (96.3%) |  |
| Alcohol |  |  |  |  |
| No | 13 (41.9%) | 2 (50.0%) | 6 (22.2%) | 0.2231 |
| Yes | 18 (58.1%) | 2 (50.0%) | 21 (77.8%) |  |
| CD4 Count (at death/censor) | |  |  |  |
| ≤200 | 16 (51.6%) | 2 (50.0%) | 13 (48.1%) | 0.9659 |
| >200 | 15 (48.4%) | 2 (50.0%) | 14 (51.9%) |  |
| Viral load % of time undetectable (at death/censor) | | |  |  |
| < 40% | 3 (9.7%) | 1 (25.0%) | 4 (14.8%) | 0.8646 |
| 40-80% | 12 (38.7%) | 1 (25.0%) | 8 (29.6%) |  |
| > 80% | 16 (51.6%) | 2 (50.0%) | 15 (55.6%) |  |
| Year of Cancer Diagnosis |  |  |  |  |
| 2010 | 2 (6.5%) | 1 (25.0%) | 7 (25.9%) | 0.0897 |
| 2011 | 6 (19.4%) | 0 (0.0%) | 6 (22.2%) |  |
| 2012 | 1 (3.2%) | 1 (25.0%) | 7 (25.9%) |  |
| 2013 | 2 (6.5%) | 0 (0.0%) | 1 (3.7%) |  |
| 2014 | 8 (25.8%) | 1 (25.0%) | 1 (3.7%) |  |
| 2015 | 3 (9.7%) | 0 (0.0%) | 3 (11.1%) |  |
| 2016 | 9 (29.0%) | 1 (25.0%) | 2 (7.4%) |  |
| Year first HIV |  |  |  |  |
| Before 1996 | 10 (32.3%) | 1 (25.0%) | 7 (25.9%) | 0.2834 |
| 1996-2000 | 8 (25.8%) | 1 (25.0%) | 7 (25.9%) |  |
| 2001-2005 | 6 (19.4%) | 2 (50.0%) | 2 (7.4%) |  |
| 2006-2016 | 7 (22.6%) | 0 (0.0%) | 11 (40.7%) |  |

**Supplemental Figure 1.** Kaplan-Meier curve for survival after cancer: a) all sites in head and neck cancer and b) for oropharyngeal cancer and non-oropharyngeal cancer by smoking, c) stage in oropharyngeal cancer and d) stage in non-oropharyngeal cancer.

a)
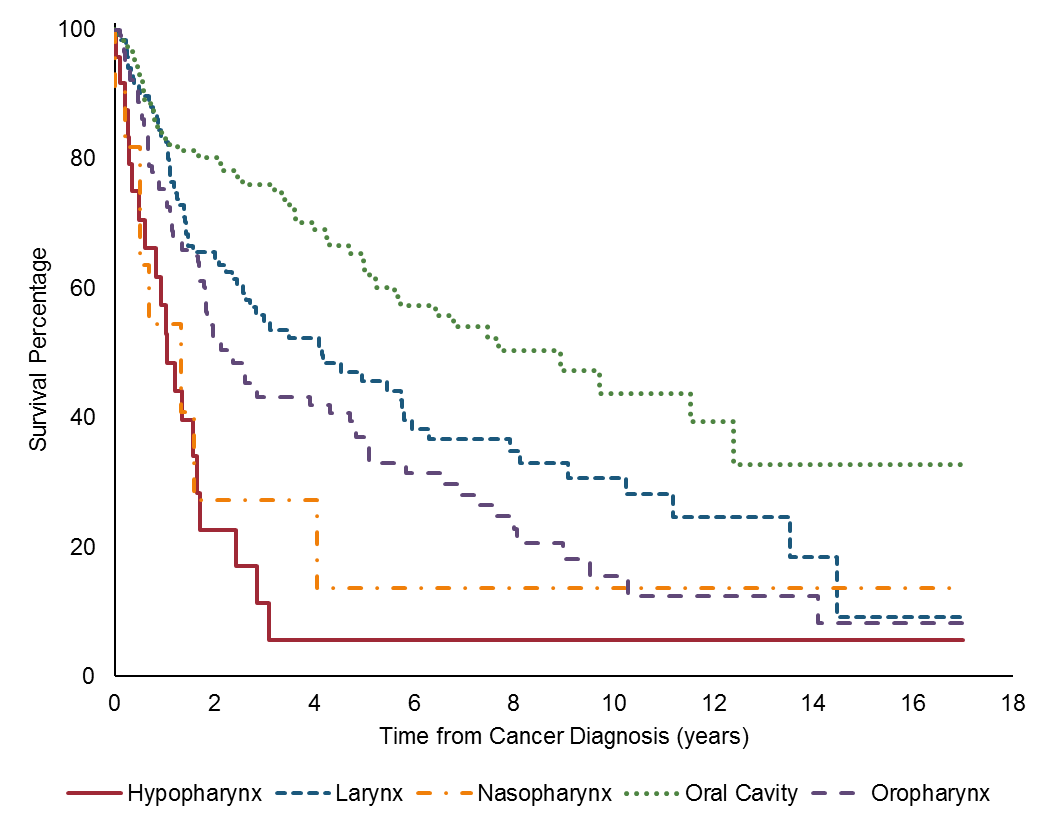


b)
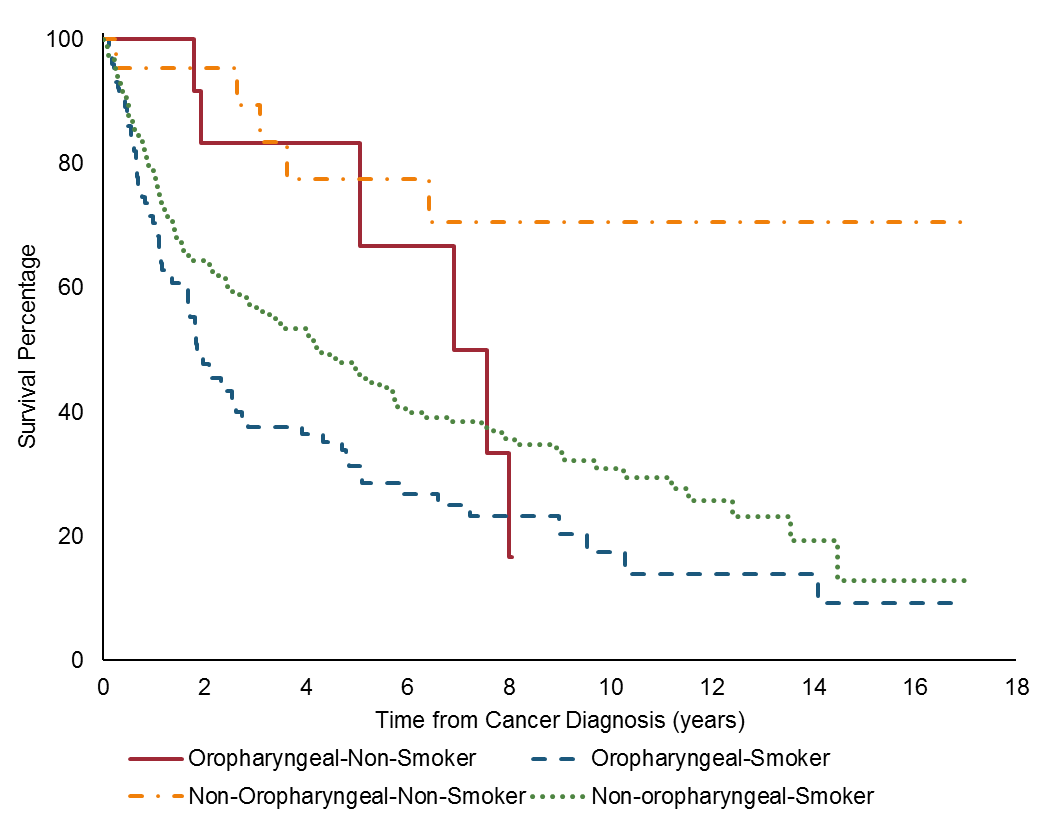


c)
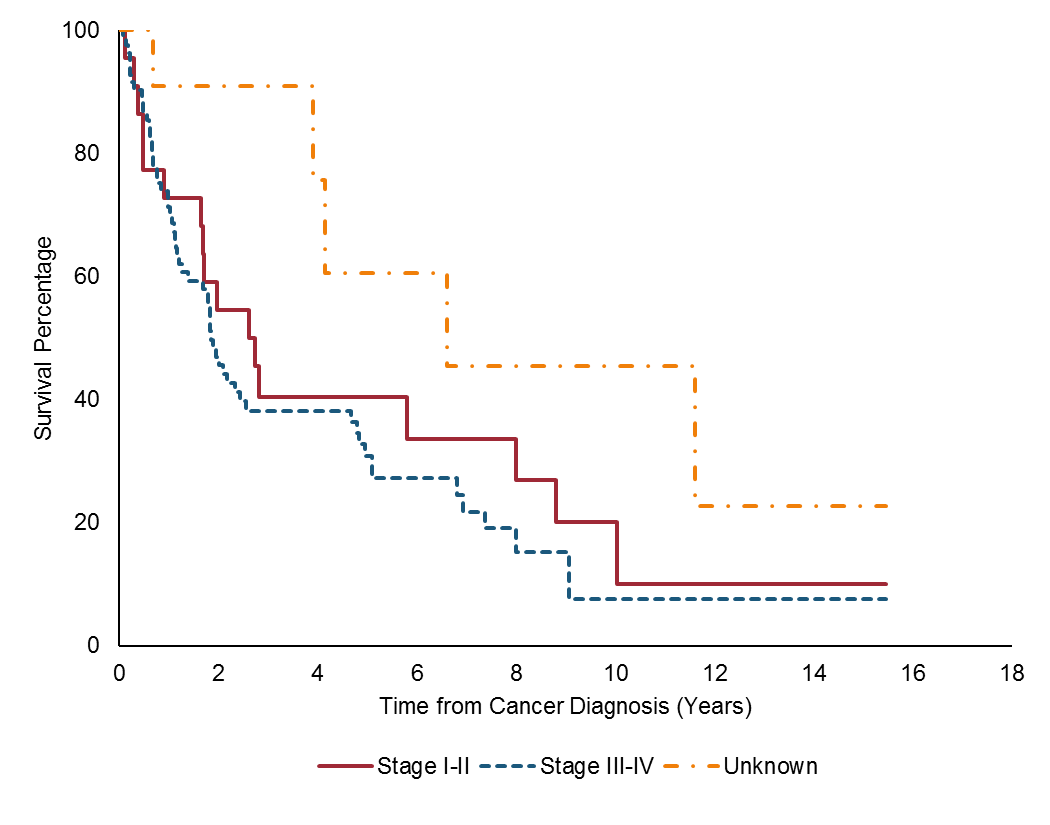


d)
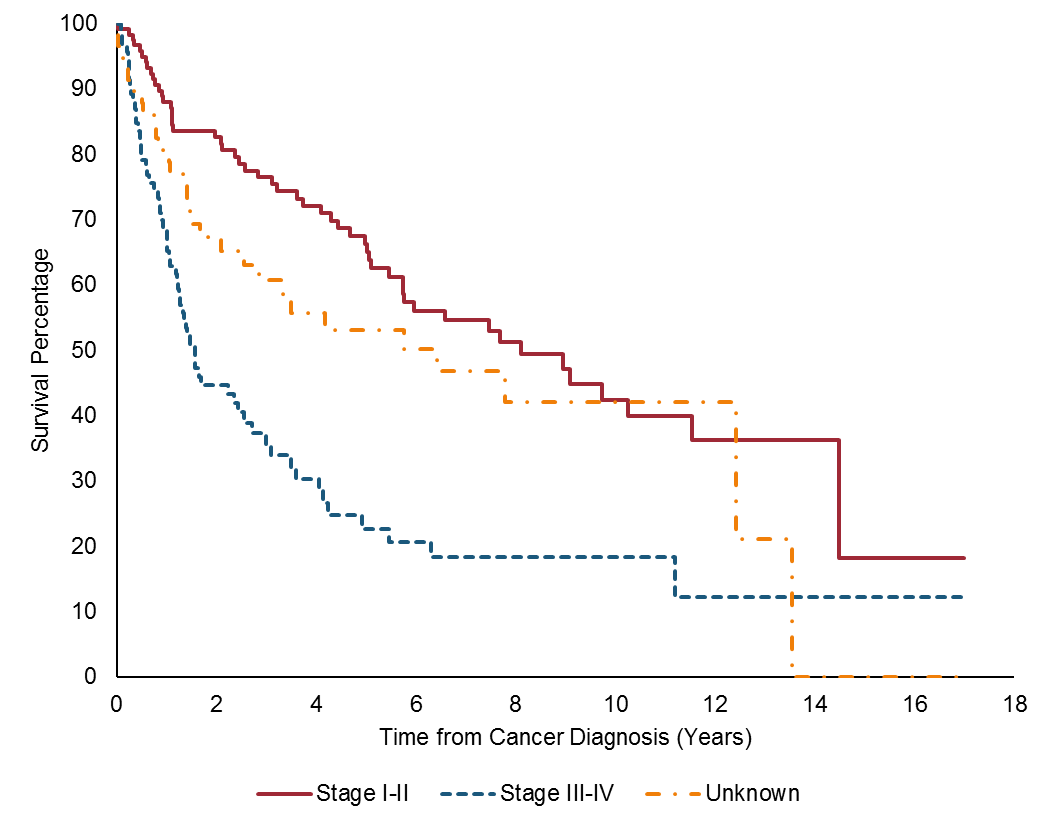

Supplement: Supplementary file 1 — Supplementary Material [file CAM4-9-9326-s001.docx]
